# Neuroinflammation and treatment resistance in major depressive disorder

**DOI:** 10.3389/fphar.2026.1838472

**Published:** 2026-06-08

**Authors:** Shingo Miyata, Yugo Ishino, Shoko Shimizu

**Affiliations:** Division of Molecular Brain Science, Research Institute of Traditional Asian Medicine, Kindai University, Sakai, Japan

**Keywords:** aging, chronic stress, glial cell functions, major depressive disorder, metabolic dysregulation, neuroinflammation, treatment resistance

## Abstract

Major depressive disorder (MDD) is increasingly recognized as a multi-system disease that extends beyond neurotransmitter dysregulation. Treatment-resistant depression (TRD), which affects approximately one-third of patients who do not achieve remission with monoaminergic antidepressants, poses a significant global challenge because of its association with a heightened risk of suicide and impaired social functioning. Low-grade chronic inflammation, a hallmark of TRD, increases blood-brain barrier (BBB) permeability. These inflammatory signals can affect the central nervous system, induce alterations in neural circuits, and contribute to depressive symptom development. A shift is necessary in the treatment of patients with TRD, moving from conventional symptom-based diagnosis to personalized medicine based on biological subtypes using inflammatory markers. In the future, complex interventions that facilitate a restorative immune environment in the brain—such as enhancing the M2 phenotype and restoring homeostasis in the nervous, immune, and endocrine systems—are anticipated to become central to next-generation antidepressant therapies. This review provides a comprehensive overview of the molecular and cellular mechanisms through which peripheral and central inflammation contribute to the pathophysiology of TRD.

## 1 Introduction

MDD contributes significantly to disability-adjusted life years (DALYs), and its global burden continues to rise as a non-communicable disease (Miyata et al., 2022; World Health Organization, 2025; Zhang et al., 2025). Research and clinical outcomes indicate that traditional monoaminergic antidepressants, including selective serotonin reuptake inhibitors (SSRIs) and serotonin-norepinephrine reuptake inhibitors, have limited efficacy (Singh et al., 2025). TRD is characterized by the failure to remit to achieve remission after ≥2 antidepressants of adequate dose/duration (∼6–8 weeks) with distinct mechanisms of action administered at appropriate dosages for a sufficient duration, typically 6–8 weeks or longer (McIntyre et al., 2023; Lucido and Dunlop, 2025). TRD affects approximately one-third of all individuals with MDD (Halaris et al., 2021). MDD and TRD are associated with a heightened risk of suicide, impaired social functioning, and high relapse rates—making them significant global health concerns (Civardi et al., 2024).

Long-term treatment of MDD has predominantly focused on increasing monoamine concentrations within the synaptic cleft. However, the occurrence of TRD indicates that MDD pathophysiology extends beyond the depletion of a single neurotransmitter (Kajumba et al., 2024). Recent advances in psychoneuroimmunology have revealed that certain individuals with MDD exhibit persistently increased peripheral levels of inflammatory markers, such as C-reactive protein (CRP), interleukin-6 (IL-6), and tumor necrosis factor-alpha (TNF-α) (Gaspersz et al., 2017; Min et al., 2023). These increases indicate a state of low-grade chronic systemic inflammation, distinct from the acute inflammation typically associated with common infections (Carrera-Bastos et al., 2025). It has been hypothesized that these mediators traverse the BBB and potentially induce depressive symptoms by modulating neural circuits (Dudek et al., 2025).

Several meta-analyses have indicated that increased levels of baseline inflammatory markers are associated with reduced treatment responsiveness (Yang et al., 2019; Osimo et al., 2020). Specifically, patients with MDD who have high-sensitivity CRP (hsCRP) levels exceeding 3 mg/L exhibit significantly diminished responses to conventional antidepressant therapy (Katamine et al., 2026). Other recent studies have indicated that elevated levels of hsCRP in patients with MDD are significantly correlated with an increased risk of suicidal behavior, including suicide attempts (Park and Kim, 2017; Miola et al., 2021).

In patients with both MDD and inflammatory diseases, inflammatory responses may impair neuroplasticity and monoaminergic reactivity (Richardson et al., 2022). Additionally, neuroimaging studies have demonstrated that inflammation reduces activity in the nucleus accumbens reward system, resulting in anhedonia and loss of pleasure, while potentially increasing activity in the amygdala and prefrontal cortex—key emotion regulation systems (Felger, 2018; Alvarez et al., 2020).

Dysregulation of inflammation, oxidative stress, and HPA axis dysfunction has been extensively documented in patients with MDD. However, accumulating evidence indicates that these abnormalities are quantitatively and qualitatively more marked in TRD than in treatment-responsive MDD. Notably, several comparative studies have demonstrated that these biomarkers do not merely reflect the pathology of depression but can serve as indicators for predicting and monitoring treatment resistance.

A significant recent development in neuropharmacology is the identification of ketamine’s efficacy as an N-methyl-D-aspartate (NMDA) receptor antagonist in the treatment of MDD. Ketamine and anti-inflammatory strategies have demonstrated efficacy in patients with established TRD (Iadarola et al., 2015; Nikkheslat, 2021). Whether early intervention with ketamine can prevent the development of TRD remains an important yet still evolving hypothesis that warrants future prospective investigations.

This review provides an overview of the molecular mechanisms underlying synaptic plasticity impairments induced by peripheral and central inflammation, functional alterations in glial cells, such as microglia and astrocytes, treatment resistance in MDD, and recent evidence-based emerging therapeutic targets.

## 2 Main text

### 2.1 Neuroinflammation and molecular and cellular pathophysiology of MDD

Neuroinflammation in MDD is not merely an accompanying phenomenon; it is an active process that significantly alters neural plasticity and neurotransmission.

#### 2.1.1 Association between peripheral cytokines and MDD

Meta-analyses have consistently demonstrated that the levels of inflammatory markers, such as IL-6, TNF-α, and CRP, are significantly increased in the blood of a subset of patients with MDD (Gaspersz et al., 2017; Min et al., 2023). Chronic stress exposure and peripheral inflammation have been reported to impair tight junction integrity in cerebral vascular endothelial cells (Menard et al., 2017). BBB disruption may enable peripheral cytokines to infiltrate the brain and impair the prefrontal cortex and hippocampus—regions responsible for emotional regulation (Blossom et al., 2025). This influx may induce behavioral alterations, including reduced motivation, loss of appetite, and sleep disturbances, which represent core symptoms of depression.

#### 2.1.2 Glial cell functional alterations and breakdown of neural circuits

Alterations in glial cells that maintain the brain microenvironment directly contribute to structural brain alterations, such as hippocampal atrophy in MDD (Miyata, 2019; Guo et al., 2022; Duarte et al., 2024; Singhal et al., 2025). Stress induces M1 microglia polarization (pro-inflammatory), realizing IL-1β/TNF-α and facilitates excessive synaptic pruning (Gao et al., 2023; Nandi et al., 2025). This reduces the neural network density essential for emotional regulation, resulting in cognitive impairment and mood instability. In inflammatory conditions, astrocytes exhibit reduced expression of glutamate transporters and impaired glutamate metabolism (Huang et al., 2025). Consequently, a persistently high concentration of glutamate in the synaptic cleft overstimulates neurons (excitotoxicity) and suppresses the production of a neuroprotective factor (brain-derived neurotrophic factor [BDNF]) (Khoodoruth et al., 2022; Nicosia et al., 2024). These findings may contribute to the reduced neurogenesis observed in patients with MDD (Dantzer and Walker, 2014).

#### 2.1.3 Transition to the kynurenine pathway

Beyond the classical serotonin deficiency hypothesis, neuroinflammation highlights the role of the kynurenine pathway in MDD (Ogyu et al., 2018). Cytokine-induced indoleamine 2,3-dioxygenase (IDO) activation diverts tryptophan from serotonin to the kynurenine pathway, yielding neurotoxic quinolinic acid (QA) (Dantzer, 2017; Hestad et al., 2022). QA is a potent NMDA agonist that generates reactive oxygen species (ROS) and induces neuronal cell death (Lugo-Huitrón et al., 2013). This pathway shift may be a significant mechanism underlying TRD, for which conventional SSRIs are ineffective.

#### 2.1.4 Metabolic and redox dysregulation

Impaired energy metabolism reduces the threshold for MDD onset. It has been widely demonstrated in cell cultures, animal models, and clinical studies that dysfunction of mitochondrial electron transport chain complexes I and IV impairs ATP production, resulting in an energy deficit that disrupts neuronal homeostasis (Jiang et al., 2024; Liu X et al., 2025; Yang, 2025). Meta-analyses studying the role of mitochondrial Complex I and Complex IV in psychiatric disorders, such as MDD, have indicated a moderate involvement of abnormal Complex I/IV patterns in MDD (Holper et al., 2019). Additionally, several studies have indicated that abnormalities in the mitochondrial respiratory chain significantly contribute to the onset of depression (Jiang et al., 2024; Zong et al., 2024; Yang, 2025). These findings suggest that energy deficiency in MDD leads to fatigue and abnormal ROS production, and that dysfunction in energy metabolism may precipitate the development of MDD.

Additionally, damage-associated molecular patterns may activate microglia, creating a self-amplifying loop between inflammation and oxidative stress (Wilkins et al., 2017; Dmytriv et al., 2024). Consequently, suppressed BDNF promoter activity reduces dendritic spine density, a loss of plasticity proposed to represent a critical threshold in MDD pathogenesis (Tartt et al., 2022).

#### 2.1.5 Aging and biological sensitivity

Epigenetic age acceleration (GrimAge or PhenoAge) observed in patients with MDD indicates chronological aging and is crucial for demonstrating the novel functions of biological sensitivity (Protsenko et al., 2021; Liufu et al., 2025). The senescence-associated secretory phenotype (SASP) may affect MDD sensitivity (Diniz, 2018). SASP consists of proinflammatory factors secreted by senescent cells, including cytokines, chemokines, growth factors, and proteases, which transfer senescence signals to adjacent cells and may contribute to the maintenance of chronic, low-grade inflammation (Lagoumtzi and Chondrogianni, 2021; Zhang et al., 2023). SASP factors, such as IL-6, IL-8, and C–C motif chemokine ligand 2, facilitate the persistent priming of quiescent microglia (Gorgoulis et al., 2019). Several studies using rodent models demonstrate that activated microglia exhibit heightened inflammatory responses, even in response to minor stressors. This heightened response may lead to cognitive decline, impaired synaptic plasticity, and accelerated neurodegeneration. (Norden and Godbout, 2013; Perry and Holmes, 2014). The concept of inflammaging in depression, indicating that patients with MDD exhibit advanced brain inflammation because of aging, constitutes both a risk factor for onset and a basis for TRD (Lorenzo et al., 2023; Carrera-Bastos et al., 2025). Additionally, they demonstrated that SASP-induced persistent inflammation negates the neuroplasticity-enhancing effects of antidepressants (Diniz et al., 2021).

### 2.2 Peripheral and central inflammation in MDD

#### 2.2.1 Pathways from peripheral inflammation to the CNS

Peripheral cytokines, such as IL-6 and TNF-α, can access the CNS through several mechanisms: (i) active transport across the BBB, (ii) passage through circumventricular organs where the BBB is less restrictive, (iii) activation of the afferent fibers of the vagus nerve, and (iv) indirect signaling mediated by endothelial cells that produce secondary mediators, such as prostaglandins and nitric oxide (Dantzer et al., 2008). Once neuroinflammatory signaling is initiated, resident microglia amplify the response by releasing additional inflammatory cytokines and reactive oxygen species, thereby establishing a self-sustaining state of inflammation in the CNS (Capuron and Miller AH, 2011; Ransohoff, 2016; Shi and Yong, 2025).

#### 2.2.2 Both peripheral and central inflammation affects MDD conditions

Recent studies do not show a simplistic, unidirectional model in MDD (Llach et al., 2025). In contrast, the relationship between peripheral and central inflammation in MDD is bidirectional and context-dependent (Beurel et al., 2020). Systemic inflammatory signals may prime microglia, whereas an activated central immune state may lower the threshold for subsequent neuroinflammatory response (Berk et al., 2013). Notably, some individuals exhibit heightened central nervous system inflammation despite the absence of significant increases in peripheral cytokines (Bloomfield et al., 2016; Carrier et al., 2020). These results suggested that central immune mechanisms may operate partially independently of peripheral inflammatory states.

#### 2.2.3 The “threshold” of inflammation for MDD

In recent years, the concept of an “inflammatory threshold” in patients with MDD has gained attention (Pastis et al., 2024). Epidemiological studies indicate that the relationship between blood cytokine concentrations and the severity of MDD symptoms is not linear; rather, there may be a threshold effect, and when this threshold is surpassed, the neuroinflammatory cascade reaches a level sufficient to induce behavioral change (Köhler et al., 2017). This threshold may reflect the capacity of compensatory anti-inflammatory pathways (e.g., IL-10 and IL-4) to counteract pro-inflammatory signaling before irreversible changes in monoaminergic neurotransmission or neural plasticity occur.

#### 2.2.4 Not all patients with systemic inflammation develop MDD

Previous studies have suggested that not all individuals with systemic inflammatory diseases, such as rheumatoid arthritis or inflammatory bowel disease, experience MDD (Matcham et al., 2013). This highlights the importance of investigating the factors that protect individuals from developing MDD. Several factors contribute to resilience or vulnerability, including (1) genetic polymorphisms in cytokine signaling pathways (e.g., IL-6 receptor variants), (2) reactivity of the HPA axis, (3) pre-existing differences in the primed state of microglia, (4) integrity of the BBB, and (5) psychosocial factors such as social support and control styles (Cattaneo et al., 2013; Miller and Raison, 2016). The concept of inflammatory sensitivity, or individual differences in neuroimmune responses, may more effectively elucidate why equivalent levels of systemic inflammation can lead to different neuropsychiatric outcomes.

### 2.3 TRD and inflammatory subtypes

#### 2.3.1 Identification of the inflammatory subtype and reduced SSRI responsiveness

Recent studies have indicated the significance of classifying patients with MDD into distinct subtypes based on biological markers, rather than adopting a uniform treatment approach for all patients with MDD (Hacimusalar and Eşel, 2018; Rovero et al., 2024). Elevated levels of inflammatory cytokines, particularly IL-6, TNF-α, and CRP, have not been consistently observed across all MDD subtypes. Patients with MDD exhibiting excessively high serum CRP levels demonstrated significantly reduced responsiveness to conventional SSRIs (Orsolini et al., 2022). Previous studies have demonstrated that individuals who do not respond to antidepressant treatment exhibit significantly higher levels of CRP and IL-6 than those who do respond, suggesting that inflammation may serve as a predictor of poor treatment outcomes rather than a general correlate of MDD (Nettis et al., 2021). Furthermore, it has been observed that patients with MDD with elevated baseline CRP levels do not respond to the tricyclic antidepressant nortriptyline or the SSRI escitalopram. Additionally, patients with TRD and high levels of inflammation display distinct neurological characteristics, such as reduced connectivity in cortico-striatal circuits, which are not present in other patients (Uher et al., 2014; Haroon et al., 2018). These studies suggest a potential mechanism by which patients may transition from MDD to TRD, contingent on their level of inflammation.

#### 2.3.2 Oxidative stress and mitochondrial dysfunction

In patients with TRD, oxidative stress levels—Closely associated with neuroinflammation—Are increased and positively associated with symptoms resistant to treatment (Winczewska et al., 2026). Excessive microglial activation releases pro-oxidants that damage lipids, proteins, and DNA by generating ROS (Smith et al., 2022). Persistent inflammation disrupts the mitochondrial electron transport chain and reduces ATP production (Yuan et al., 2022). This disruption impairs cerebral energy metabolism, contributing to characteristic depressive symptoms, such as cognitive dysfunction (brain fog; Jiang et al., 2024). TRD is characterized by reduced levels of antioxidants, such as glutathione, and a significant increase in markers of oxidative damage, such as 8-hydroxy-2′-deoxyguanosine (Winczewska et al., 2026). Previous findings indicate that these markers are excessively elevated in patients with TRD compared to those who respond to antidepressant treatments. Additionally, oxidative stress indicators tended to normalize in states responsive to antidepressants, whereas such normalization was absent in patients with TRD (Lindqvist et al., 2017).

#### 2.3.3 Hypothalamic-pituitary-adrenal (HPA) axis abnormalities

The HPA axis plays a central role in stress response, and the immune system maintains equilibrium through negative feedback (Tohyama et al., 2015; Miyata et al., 2022; Shimizu et al., 2024). Hyperactivity of the HPA axis, as evidenced by a lack of suppression in the dexamethasone suppression test and an elevated cortisol awakening response, has been consistently associated with MDD (Juruena et al., 2009; 2010). However, a systematic comparison of the functions of GR and the mineralocorticoid receptor between patients with TRD and treatment-responsive MDD revealed that GR resistance is significantly greater in patients with TRD (Pariante and Miller, 2001). These findings suggest that GR resistance may be directly related to the mechanism by which MDD progresses to become TRD (Pace et al., 2007; Coutinho and Chapman, 2011). Cortisol binds to GRs to suppress inflammation. However, chronic exposure to inflammatory cytokines reduces the efficacy of GR signaling, a phenomenon known as GR resistance. Research on both clinical studies and rodent models indicates that glucocorticoid resistance, resulting from GR dysfunction, may exacerbate inflammation and activate the corticotropin-releasing hormone (CRH) and sympathetic nervous systems (Pace et al., 2007). Additionally, studies in mice have demonstrated that following lipopolysaccharide (LPS) administration, there is an excessive inflammatory response, prolonged corticosterone elevation, and pathological behavior. Moreover, findings from both clinical and rodent studies have revealed that inflammatory signaling pathways, such as the NLRP3 pathway, activate caspase-1, which subsequently cleaves GR, thereby contributing to glucocorticoid resistance (Silverman and Sternberg, 2012; Miller and Raison, 2016). This condition may inhibit hippocampal neurogenesis, resulting in persistent cognitive decline and impaired emotional regulation in patients with TRD.

Furthermore, elevated hsCRP, an inflammatory marker, levels in patients with MDD are associated with an increased risk of suicide, encompassing both suicide attempts and severe suicidal ideation, regardless of the severity of MDD (Park and Kim, 2017; Miola et al., 2021). This suggests that elevated hsCRP levels may contribute to suicidal behavior and could potentially serve as a biological trait marker for it.

### 2.4 Latest neuropharmacological targets

Interventions targeting the glutamate system may rapidly activate neurons and restore neuroplasticity. As previously discussed, inflammatory conditions in patients with MDD may reduce glutamate reuptake, leading to excitotoxicity (Haroon et al., 2017). Consequently, regulating glutamate receptor signaling has recently become a research focus in MDD.

#### 2.4.1 Ketamine and esketamine

Several studies using rodent models have demonstrated that ketamine and esketamine primarily exert rapid and sustained antidepressant effects by activating the mTOR pathway through NMDA receptor (NMDAR) antagonism. This process enhances synaptic signaling proteins, leading to increased formation and functional maturation of dendritic spine synapses in the prefrontal cortex (Cui et al., 2019; Luscher et al., 2020; Merlo et al., 2023; Seshadri et al., 2024; Silva et al., 2025). These rapid antidepressant effects have also been confirmed in numerous clinical trials (Vekhova et al., 2025). Additionally, recent clinical studies have reported their neuroprotective effects under inflammatory conditions (Mohammad et al., 2022). This approach may rapidly reduce the depressive effects of inflammatory cytokines (Wang et al., 2025).

A key pathological condition in which such neuroprotection is relevant is neuroinflammation-driven excitotoxicity. Neuroinflammation facilitates the release of excitotoxic glutamate through multiple convergent mechanisms. First, activated microglia and astrocytes secrete inflammatory cytokines, including IL-1β, TNF-α, and IL-6, which inhibit the function of glutamate reuptake transporters, particularly GLT-1/EAAT2, resulting in glutamate accumulation within the synapse (Tilleux and Hermans, 2007; Zhang et al., 2022). Concurrently, the activation of inflammasomes, NLRP3, and oxidative stress has been documented to further elevate extracellular glutamate concentrations, initiating a self-perpetuating excitotoxic cascade (Chiarini et al., 2023; Xu et al., 2025).

This extracellular glutamate accumulation leads to the pathological overactivation of NMDARs, especially those containing the NR2B subunit located in the extrasynaptic region. Persistent activation of extrasynaptic NMDARs triggers a well-established cell death cascade characterized by Ca^2+^ overload, mitochondrial dysfunction, and activation of calpains and caspases, ultimately resulting in neuronal apoptosis and necrosis (Hardingham and Bading, 2010; Zhang et al., 2020). This mechanism has been confirmed in several neuroinflammatory models, including those of septic encephalopathy, traumatic brain injury, and ischemia (Bhatt et al., 2023; Wang et al., 2021).

Ketamine and esketamine, as non-competitive NMDAR antagonists, preferentially inhibit excessively activated extrasynaptic NMDARs, thereby directly obstructing this excitotoxic cascade, reducing pathological Ca^2+^ influx, and attenuating downstream cell death signaling (Choudhury et al., 2021; Silva et al., 2025). Moreover, subanesthetic doses of ketamine have been found to concurrently activate the mTOR complex 1 pathway via disinhibition of α-amino-3-hydroxy-5-methyl-4-isoxazole propionic acid (AMPA) receptor–BDNF–TrkB signaling, promoting synaptogenesis, restoring synaptic protein synthesis, and exerting anti-apoptotic effects (Li et al., 2010; Zanos et al., 2018). This dual mechanism, NMDAR blockade and mTOR-mediated neuroprotection, constitutes the mechanistic foundation for the neuroprotective potential of ketamine and esketamine in neuroinflammatory conditions, underscoring their broader therapeutic significance beyond traditional antidepressant applications.

#### 2.4.2 Purinergic receptor P2X ligand-gated ion channel 7 (P2X7) receptor antagonists, COX-2 inhibitors, such as celecoxib, and TNF inhibitors

Drug repositioning is a promising approach for treating MDD. Previous studies have demonstrated that in patients with increased blood CRP or TNF-α levels, the combined use of antidepressants with P2X7 receptor antagonists and COX-2 and TNF inhibitors enhances remission rates (Müller, 2019; Drevets et al., 2022; Liu J et al., 2025; Yin et al., 2025).

#### 2.4.3 Repositioning anti-inflammatory drugs for MDD treatment

In recent years, accumulating evidence has supported the use of anti-inflammatory and metabolism-targeted pharmacological interventions for MDD and TRD (Miller and Raison, 2016). In specific cohort studies of patients with MDD, consistent findings have indicated elevated levels of inflammatory cytokines, such as interleukin-6 (IL-6), tumor necrosis factor-alpha (TNF-α), and C-reactive protein (CRP), along with microglial activation and dysregulation of the HPA axis (Dantzer et al., 2008; Goldsmith et al., 2016). These observations suggest that anti-inflammatory and metabolic agents targeting immune regulatory mechanisms may exert antidepressant effects.

Metformin has been reported to exhibit antidepressant-like properties not only through its metabolic actions but also by suppressing neuroinflammation via AMPK and modulating the gut-brain axis (Ai et al., 2020; Maciejczyk and Blecharz-Klin, 2025). Statins, as evidenced by both observational studies and randomized controlled trials, have demonstrated adjunctive antidepressant effects by reducing CRP and IL-6 levels and normalizing serotonergic transmission (Köhler et al., 2014; Gougol et al., 2015; Salagre et al., 2016). Statins lower lipid levels, suppress pro-inflammatory cytokine production, and enhance endothelial function, thereby protecting the BBB (Morofuji et al., 2022; Kim et al., 2025). Furthermore, metformin and statins have the potential to inhibit pro-inflammatory microRNAs (miRNAs) or augment neuroprotective miRNAs. Therapies can be developed that fundamentally rebalance the immune balance in the brain (Docrat et al., 2021; Loan et al., 2024).

Among the targeted biological agents, IL-6 receptor antagonists, such as tocilizumab and satralizumab, have shown partial efficacy in treating the inflammatory depression phenotype (Raison et al., 2013; Kappelmann et al., 2018; Ting et al., 2020; Mancuso et al., 2023). GR antagonists, particularly mifepristone, have therapeutic potential for MDD and TRD by normalizing the hyperactivity of the HPA axis (DeBattista et al., 2006; Blasey et al., 2009; Kruizinga et al., 2021). Additionally, JAK and TNF-α inhibitors are garnering attention as potential treatment candidates for patients with elevated baseline inflammatory markers who do not respond adequately to conventional antidepressants (Strawbridge et al., 2015; Haroon et al., 2018; Chamberlain et al., 2019). Multiple meta-analyses have demonstrated that COX-2 inhibitors, such as celecoxib, and TNF inhibitors enhance remission rates in patients with increased blood CRP or TNF-α levels when used alongside antidepressants (Bortolato et al., 2015; Uzzan and Azab, 2021).

#### 2.4.4 Next-generation novel targets for MDD treatment

Identifying novel molecular targets is crucial to achieve more precise emotional regulation in patients with MDD. Specific miRNAs, such as miR-155 and miR-124 simultaneously regulate the expression of numerous genes and act as switches in neuroinflammation (Gaudet et al., 2018; Asl et al., 2025). Developing novel molecular targets for MDD treatment may modulate the brain’s immune balance by inhibiting pro-inflammatory miRNAs or increasing neuroprotective miRNAs. Additionally, opioid receptor inactivation contributes to motivational deficits (Puryear et al., 2020). It is well known that chronic inflammation suppresses the brain’s reward and dopaminergic systems, resulting in significant apathy. Therefore, kappa opioid receptor antagonists and mu opioid receptor modulators may specifically ameliorate inflammation-induced damage to the reward system, thereby enhancing anhedonia and reducing motivation (Cuitavi et al., 2023; Dalefield et al., 2022).

## 3 Conclusion

Patient heterogeneity is a major challenge in MDD treatment. Therefore, transitioning from traditional symptom-based diagnosis to personalized medicine based on biological evidence is necessary. Mild chronic inflammation (low-grade inflammation) is observed in approximately 30%–40% of all patients with MDD (Zalli et al., 2016; Bergmans and Malecki, 2017; Mehdi et al., 2023). Increased levels of biomarkers, such as serum CRP, IL-6, or soluble TNF receptors, can be used to pre-identify an inflammatory subtype, enabling the optimization of MDD treatment (Elbakary et al., 2025). For patients with MDD with increased inflammatory profiles, early selection of NMDA receptor modulators (ketamine and esketamine) or specific anti-inflammatory therapies may help prevent the transition to TRD. Therefore, the association between neuroinflammation and MDD has progressed beyond a theoretical hypothesis and is increasingly recognized as the principal pathophysiology underlying TRD (Bhatt et al., 2023). BBB dysfunction, glial cell polarization, shifts in the kynurenine pathway, and HPA axis hyperactivity do not occur independently; rather, they amplify one another, forming inflammatory cascades in patients with MDD (Bose et al., 2026). The limitations of existing monoamine reuptake inhibitors, such as SSRIs, are inadequate to repair inflammation-induced structural and metabolic damage, including synaptic loss and quinolinic acid toxicity (Richardson et al., 2022; O'Regan et al., 2026). Additionally, novel agents and interventions targeting multiple mechanisms, such as P2X7 receptor antagonists, miRNA-targeted therapies, and neuroprotection through metabolic pathways, such as metformin, are essential for enhancing remission rates in patients with TRD (Cai et al., 2024; Aljabali et al., 2025; Liu J et al., 2025).

It is pivotal to acknowledge that numerous biological abnormalities discussed in this paper, such as inflammation, HPA axis dysfunction, and oxidative stress, are not exclusive to TRD but are observed throughout the entire spectrum of MDD. Nevertheless, converging evidence from longitudinal biomarker studies, treatment-stratified cohorts, and prospective clinical trials supports the model that these mechanisms function along a continuum of disease severity. TRD is not merely a more severe variant of MDD symptoms but represents a biologically distinct state. As demonstrated in prospective studies, including relevant substudies of GENDEP and STAR*D, the relationship between these biomarkers and treatment non-response is predictive rather than merely associative, thereby strengthening the inference that these pathways causally contribute to treatment resistance (Krishnan and Nestler, 2008; Trivedi et al., 2006). Future studies engaging interventional designs (e.g., anti-inflammatory augmentation therapies in high-CRP TRD versus low-CRP MDD) are essential to establish the specificity of these mechanisms.

**FIGURE 1:**
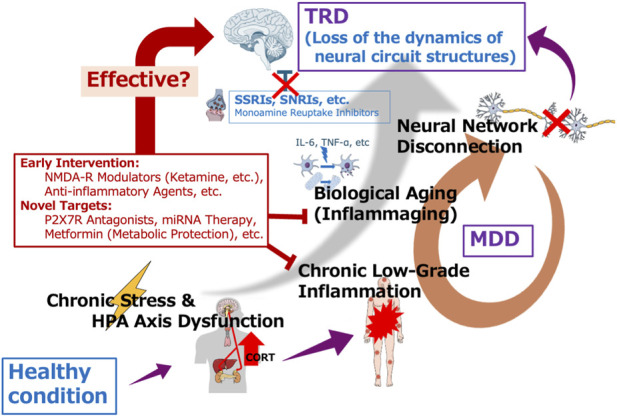
Expected mechanism of leading to TRD. This figure indicates the biological progression from a chronic stress state to TRD. It is thought that chronic inflammation initiates inflammatory aging, leading to physical disconnection within neural networks. This disconnection may result in a diminished efficacy of treatment with monoamines.

Future research should comprehensively elucidate the relationship between neuroinflammation, summarized in this review, as a molecular mechanism contributing to TRD symptoms, and functional alterations in neurons, oligodendrocytes/myelin, astrocytes, microglia, and the vascular system. These insights may be correlated with effective treatment (Figure 1).
